# Evaluation of Growth Performance, Nitrogen Balance and Blood Metabolites of Mutton Sheep Fed an Ammonia-Treated Aflatoxin B1-Contaminated Diet

**DOI:** 10.3390/toxins14050361

**Published:** 2022-05-23

**Authors:** Meimei Zhang, Peixin Jiao, Xue Wang, Youran Sun, Gege Liang, Xiaolai Xie, Yonggen Zhang

**Affiliations:** College of Animal Science and Technology, Northeast Agricultural University, Harbin 150030, China; mmzhang12@126.com (M.Z.); peixin.jiao@neau.edu.cn (P.J.); wangxzxc@163.com (X.W.); syr1000@126.com (Y.S.); lgg13422166268@126.com (G.L.)

**Keywords:** aflatoxin B1, ammoniation, blood metabolites, growth performance

## Abstract

Experiments were conducted to evaluate the effects of an aflatoxin B1 (AFB1)-contaminated diet treated with ammonia on the diet detoxification and growth performance, nutrient digestibility, nitrogen utilization, and blood metabolites in sheep. Twenty-four female mutton sheep with an initial body weight of 50 ± 2.5 kg were randomly assigned to one of three groups: (1) control diet (C); (2) aflatoxin diet (T; control diet supplemented with 75 μg of AFB1/kg of dry matter); and (3) ammoniated diet (AT; ammoniated aflatoxin diet). The results showed decreases (*p* < 0.05) in average daily feed intake, nutrient digestibility of dry matter, crude protein and ether extract, and retained nitrogen, and an increase (*p* < 0.05) in urine nitrogen excretion in sheep fed diet T compared with those fed the other diets. In comparison to C and AT, feeding T decreased (*p* < 0.05) the concentrations of total protein, immunoglobulin A, immunoglobulin G, immunoglobulin M, superoxide dismutase, and total antioxidants and increased (*p* < 0.05) the concentrations of alanine amino transferase, malondialdehyde, and interleukin-6. In summary, ammonia treatment has the potential to decrease the concentration of AFB1 and alleviate the adverse effects of AFB1.

## 1. Introduction

Aflatoxin is a low-molecular-weight secondary metabolite produced mainly by Aspergillus flavus and Aspergillus parasiticus. It is commonly found in food and feed and is considered to be the most toxic mycotoxin [1,2]. Aflatoxin B1 (AFB1), aflatoxin B2 (AFB2), aflatoxin G1 (AFG1), and aflatoxin G2 (AFG2) are natural forms of aflatoxin [3]. Among all aflatoxins, AFB1 is the most prevalent and the most toxic and is known to contaminate corn, sorghum, rice, peanuts, and cottonseed before and during crop harvesting, storage, and processing and manufacturing [4,5]. AFB1 has a wide variety of effects on animals: it can decrease feed intake, inhibit animal growth and development, reduce immune function, and cause oxidative stress [6,7]. AFB1 also affects protein synthesis, causing metabolic disorders, which in turn affect animal body functions [8]. Chronic exposure to aflatoxins in the diets of farm animals can decrease appetite and antioxidant capacity and impair liver and kidney function [9,10,11]. Ogunade et al. [12] found that ingestion of AFB1-contaminated feed affects the growth performance and immune status of dairy cows. Studies have shown that aflatoxins can reduce the body weight and growth rate and affect the mineral metabolism of young lambs [13,14,15].

Many methods have been used to remove AFB1 from food or feed, including ammoniation, thermal inactivation, irradiation, fermentation, and nixtamalization [16]. Studies have demonstrated that the ammoniation of aflatoxin is an effective and safe method for the detoxification of aflatoxin in animal feed [17]. Ammonia can open the lactone ring of AFB1, followed by the formation of an ammonium salt with the resulting hydroxy acid, thereby reducing the toxin content [18].

Previous research focused mainly on the detoxification of AFB1 by aqua-ammonia or NH_4_OH (liquid). However, the detoxification of AFB1 by ammonia gas is rarely reported. Moreover, to the best of our knowledge, literature on the evaluation of ammoniated agricultural products added to mutton sheep diets is scarce. In this study, we hypothesized that ammoniation could reduce aflatoxin content in the diet, and sheep fed an ammoniated AFB1 diet did not affect the growth performance and nutrient digestibility of animals. Therefore, the objective of this study was to investigate the effects of ammoniation on the destruction of aflatoxins in diets and compare the growth performance, nutrient digestibility, N utilization, and blood profile parameters in mutton sheep fed either AFB1-contaminated diets or AFB1-contaminated diets detoxified by ammoniation.

## 2. Results

### 2.1. Aflatoxin Detoxification

The AFB1 content results are shown in Table 1. The content of AFB1 in the ammoniation group was significantly lower than that in the untreated group (*p* < 0.05). The AFB1 content in the 0.3 MPa group was the lowest, at 360.13 μg/kg. There was no significant difference between the 0.3 MPa and 0.2 MPa groups (*p* > 0.05), but both levels were significantly lower than those of the 0.1 MPa and untreated groups (*p* < 0.05). The AFB1 content in the 3 h group was the lowest, 248.15 μg/kg, which was significantly lower than that in the untreated group (*p* < 0.05). There was no significant difference between the 1 h and 2 h groups (*p* < 0.05). Moreover, 0.3 MPa in the 3 h group had the lowest AFB1 content (65.9 μg/kg) among the treatments. The interaction of time and pressure had a significant influence on AFB1 content (*p* < 0.05).

### 2.2. Growth Performance and Nutrient Digestibility

As shown in Table 2, the ADG tended to decrease (0.05 < *p* < 0.10), and the DMI was lower (*p* < 0.01) in T than in the other treatment groups; there was no significant difference in FCR among the three groups (*p* > 0.05). Compared with diet C and diet AT, diet T reduced (*p* < 0.05) the apparent digestibility of DM, CP, and EE.

### 2.3. Nitrogen Utilization

Table 3 shows the results of nitrogen utilization. The N intake, retained N, and proportion of N intake were lower (*p* < 0.05) in T than in the other treatment groups, and urine nitrogen excretion and proportion of N intake were higher (*p* < 0.05) in T than in the other treatment groups. Compared to C, feeding AT increased (*p* < 0.05) fecal nitrogen excretion and the proportion of N intake.

### 2.4. Blood Metabolites

The results of blood biochemical indicators are shown in Table 4. The contents of TP and ALB in sheep fed the T diet were lower (*p* < 0.05) than those in sheep fed the C and AT diets; however, the contents of ALT and AST were greater (*p* < 0.05) in T than in the other treatment groups. There was no significant difference in the contents of ALP, TG, TC, BUN, and Glu among the three groups (*p* > 0.05).

### 2.5. Antioxidant Index

In comparison to C and AT, feeding T reduced (*p* < 0.05) the activities of SOD, T-AOC, and GSH-PX and increased (*p* < 0.01) the concentration of MDA (Table 5). The concentrations of IgA, IgG, IgM, and IL-4 in sheep fed the T diet were lower (*p* < 0.05) than those in sheep fed the C and AT diets, and the concentration of IL-6 in sheep fed the T diet was greater (*p* < 0.05) than those in sheep fed the C and AT diets. There was no significant difference in the contents of IL-2 among the three groups (*p* > 0.05).

## 3. Discussion

### 3.1. Aflatoxin Detoxification

Aflatoxin B1 is a derivative of dihydrofuroxazeo-ketone; its difuran ring is the basic toxic unit, and oxazeo-ketone is associated with strong toxicity and cancer [19]. Under the action of ammonia gas, the lactone ring of aflatoxin B1 breaks and dehydroxylates, forming ammonium salt and eliminating its toxicity [18]. Allameh et al. [20] reported that treating grains with ammonia could reduce aflatoxin levels from 1000 or 2000 ppb to below 10 ppb, which is a viable approach for the detoxification of aflatoxins. In this study, the longer ammoniation time and higher pressure caused a more significant detoxification effect, which is consistent with Nyandieka et al. [21] and Norred [22]. Nyandieka et al. [21] treated AFB1 with ammonia at different concentrations (0.25%, 0.5%, 1.0%, 1.5%, and 2.0%) under high pressure (2 bar) and low pressure (atmospheric pressure) and found that ammonia more effectively detoxified aflatoxins under high pressure and a high concentration than under atmospheric or low pressure.

### 3.2. Growth Performance and Nutrient Digestibility

It has been reported that aflatoxin B1 can cause decreases in the growth performance of many species, such as cattle, sheep, and pigs [23,24]. The level of aflatoxin in the diet affects the weight gain rate. For every mg/kg increase in aflatoxin in the diet, the growth rate of pigs and broilers decreases by 16% and 5%, respectively [25]. Moreover, a study reported that supplementation with AFB1 at a dose of 196.8 μg/kg could reduce the growth performance of meat ducks [26]. In this study, mutton sheep fed with AFB1 diets decreased ADG and DMI compared with the control diet, which is consistent with previous studies by Zhang [5], who reported a lower ADG in the diets containing AFB1 fed to calves, although the difference was not significant when compared with the control. The possible mechanism by which AFB1 influences growth performance might be attributed to the downregulation of genes related to energy production and fatty acid metabolism, resulting in impaired protein synthesis and an inability to mobilize fat [27,28].

Studies have shown that pigs fed diets contaminated with AFB1 can significantly reduce the nutrient digestibility of DM and EE [29]. Kermanshahi et al. [30] reported that feeding AFB1 at 0.8 and 1.2 mg/kg significantly decreased the nutrient digestibility of DM in broiler chickens; however, the digestibility of EE and CP was not affected by AFB1. In this study, AFB1 reduced the digestibility of DM, CP, and EE in mutton sheep. The reason may be that aflatoxin can stimulate the gastrointestinal tract, causing pathological changes and gastric mucosal damage, thus affecting the intestinal function of livestock and decreasing nutrient digestibility [31]. Moreover, Wan et al. [32] found that the villus height and the villus/crypt ratio of the duodenum, jejunum, and ileum decreased linearly with increasing concentrations of AFB1-contaminated corn in the diet, affecting the absorption of nutrients and reducing their digestibility. Unfortunately, intestinal histology was not measured in this study, and additional studies are needed to study the effects of aflatoxin on the intestinal histology of sheep.

### 3.3. Nitrogen Balance

The level of nitrogen intake in ruminants is affected mainly by dietary protein and is also a major factor affecting nitrogen deposition [33]. The nitrogen deposition rate can reflect the digestion and absorption of dietary protein in the body and the quality of crude protein in the diet. In this study, the N intake and retained N in sheep fed with AFB1 diets significantly decreased, and the urine N significantly increased, indicating that AFB1 reduces N utilization. N utilization is strongly affected by N intake [34]. Thus, the decrease in N utilization in sheep fed with AFB1 diets could also be related to the reduction in N intake in the current study. Our results are consistent with the findings of Lynch et al. [35], who reported a decrease in the nitrogen utilization of calves along with increased urinary nitrogen loss due to aflatoxin exposure. Moreover, the decreased digestibility of CP in this study indicated that AFB1 could limit the absorption of amino acids in the small intestine, thereby increasing urinary N levels. The level of fecal N in the ammoniation group was significantly greater than in the control and AFB1 groups, which may be associated with the increase in N intake resulting from ammonia treatment.

### 3.4. Blood Metabolites

Serum AST and ALT activities are sensitive indicators of liver damage and can reflect the health status of livestock and poultry [36]. In this study, AFB1 exposure significantly increased the serum activities of ALT and AST in mutton sheep, indicating that AFB1 causes liver damage [37]. The reason may be partly related to AFB1 binding to DNA to form the AFB1-8,9-epoxide complex, which is a precursor of cell necrosis and carcinogenicity [38]. Consistently, Harvey et al. [39] reported that the serum enzymatic activities of ALP and AST increased in barrows consuming 3 mg of AFB1/kg of feed. In addition, Zhang et al. [5] found that AFB1 exposure increased the plasma AST and ALP levels of calves, which is consistent with the current findings. However, no significant differences were observed for ALT and AST activities in the AT group compared with the C group, suggesting that ammoniation reduced the fluctuation in plasma enzyme activity induced by AFB1. This is consistent with the results of Allameh et al. [20], who found that ammonia treatment could reverse the biochemical alterations caused by AFB1 in broilers. TP and ALB are synthesized mainly by the liver and are important indicators reflecting the absorption and metabolism status of proteins in the body as well as the protein synthesis capacity in the liver [40]. When the liver is injured, protein synthesis is also affected. The decreased levels of TP and ALB in serum can be used as an indicator for diagnosing AFB1 poisoning [26]. A study reported that mutton sheep fed 1 mg/kg body weight AFB1 tended to have increased serum AST and ALT contents and significantly reduced TP content. Consistently, we noted that AFB1 significantly reduced the plasma concentrations of TP and ALB in this study. However, the administration of 50 μg of AFB1/kg to dairy goats did not affect serum TP, AST, or ALT contents. The inconsistent results could be due partly to the different dosages of AFB1 in the diet.

### 3.5. Antioxidants and Immune Function

Oxidative stress caused by the imbalance of reactive oxygen species (ROS) could cause tissue damage and loss of normal cell functions in animals [41]. Studies have shown that AFB1 can increase ROS production and decrease the activities of SOD, CAT, and GSH-Px in rats [7]. In addition, Shi et al. [42] suggested that AFB1-contaminated diets decreased the activities of serum SOD and increased the concentration of serum MDA in broiler chicks. Fu et al. [11] reported a decrease in the activities of serum T-SOD, GSH-Px, and CAT in weaned piglets with diets including AFB1. In agreement with these studies, a significant influence of AFB1 on antioxidant enzyme activities (SOD, T-AOC, and GSH-Px) and MDA content was observed, which may be related to AFB1 inducing oxidative stress and liver abnormalities [43]. However, the lower plasma MDA and greater antioxidant enzyme activities in sheep fed the ammoniated diet demonstrated that ammoniation reduces aflatoxin levels, thereby reducing oxidative stress in mutton sheep.

Immunoglobin A, Immunoglobin M, and Immunoglobin G are important immune indices that are produced mainly by the transformation of B lymphocytes into plasma cells and participate in humoral immunity [44]. In this study, the contents of IgA, IgG, and IgM in the AFB1 group were significantly lower than those in the control and ammoniation groups, indicating that AFB1 could decrease humoral immune function. The effects of AFB1 on immune function could be explained by the binding of AFB1 to DNA or RNA, which leads to disordered nucleic acid metabolism, affects the development of the thymus, reduces the number of lymphocytes, and suppresses the cellular and humoral immunity of animals, thus affecting the health of animals and reducing their immunity [45]. Wan et al. [32] showed that the administration of 50 μg/kg AFB1 to ducks decreased the IgA, IgG, and IgM contents of serum, which is consistent with our findings.

Cytokines are secreted by both immune and nonimmune cells, and they are important components of cellular immunity that play important roles in the immune system [46]. IL-2, IL-4, and IL-6 are cytokines produced by external stimulation of lymphocytes and are important in the immune response and inflammatory regulation [47]. The toxic effects of AFB1 lead to massive production of free radicals and potentially cause oxidative stress, stimulating the production of proinflammatory cytokines such as TNF-α, IL-1β, and IL-6 [48,49]. In this study, AFB1 increased the content of the proinflammatory cytokine IL-6 and decreased the content of the anti-inflammatory cytokine IL-4, indicating that aflatoxins could induce immune inflammatory responses. Our result is consistent with the findings of studies conducted by Ellinger-Ziegelbauer et al. [50] and Qian et al. [51], who reported that AFB1 could enhance IL-6 expression and decrease IL-4 expression in mice. The expression of these cytokines was unaffected in sheep fed an ammonia-treated diet, suggesting that the detoxification of AFB1 by the ammonia treatment of grains is probably responsible for recovering the cytokine alterations in sheep.

## 4. Conclusions

Feeding AFB1-contaminated diets decreased growth performance; the apparent digestibility of DM, CP, and EE; and nitrogen utilization in mutton sheep. Additionally, dietary AFB1 decreased the antioxidant status and immune function in the blood. However, ammonia treatment alleviated the adverse effects caused by the toxin. The results indicate that ammonia detoxification has the potential to remove aflatoxin from diets and has no side effects.

## 5. Materials and Methods

The study received approval from the institutional Animal Care Committee, Northeast Agricultural University (Harbin, China), and all experimental procedures were performed in accordance with the university’s guidelines for animal research.

### 5.1. Preparation of AFB1

The AFB1 used in the study was produced by cultivating toxigenic Aspergillus flavus (No. 3.4409), which was obtained from the China General Microbiological Culture Collection Center (Beijing, China). The ingredients used as a carrier for AFB1 were as follows: 50% corn kernels, 25% rice, and 25% barley. After being mixed thoroughly, the grain carrier was adjusted to 35% moisture, placed in 2-L gas-permeable cap polyethylene plastic buckets, and sterilized by autoclaving at 121 °C for 20 min. The carrier was inoculated with A. flavus and incubated at 25 °C for 4 wks.

### 5.2. Ammoniation of Cereals Containing AFB1

Ammoniation was completed in a closed ammoniation tank. The ammoniating test equipment was independently designed and developed by the research group of Northeast Agricultural University. The equipment consisted of an ammonia gas tank with a capacity of 170 kg, an ammonia vapor generator (2 L), and an electronic digital controlling box that shows pressure and time; the safe working pressure of the equipment is 0.3 MPa. The ammoniation conditions were 0.1, 0.2, and 0.3 MPa and 1, 2, and 3 h, respectively. The concentrations of AFB1 in all samples were analyzed by the HPLC method described by the China Feed Industry Standardization Technical Committee (2014).

### 5.3. Animals, Experimental Design, and Diets

Twenty-four female crossbred (German Mutton Merino × Northeast fine wool sheep) mutton sheep (initial body weight (BW), 50 ± 2.5 kg, eight months of age) were randomly assigned to 1 of 3 groups (*n* = 8): (1) control diet; (2) aflatoxin diet (T; control diet supplemented with 75 μg of AFB1/kg of dry matter); and (3) ammoniated diet (AT; ammoniated aflatoxin diet). Sheep were housed individually in pens with free access to water and fed ad libitum twice a day at 8 am and 5 pm. Sheep were fed for 44 days, including 14 days of adaptation to diets and 30 days of data and sample collection. The dietary ingredients and chemical composition are shown in Table 6.

### 5.4. Data Collection and Sampling

Feed offered and refused for individual sheep was recorded daily during the collection period to calculate dry matter intake. The sheep were weighed at the beginning of the study (before AFB1 and ammoniated AFB1 administration) and at the end of the study to calculate the average daily gain (ADG). Samples of individual feed ingredients, orts, and diets were collected weekly during the experimental period, stored at −20 °C and later pooled by sheep and days.

All sheep were housed in individual metabolism cages (1.2 m × 1.5 m) during the last 7 days of the experiment to determine apparent total tract digestibility and nitrogen metabolism. The fecal and urea samples were collected using a plastic screen and container sitting underneath the cage. The fecal output from each sheep was collected and quantified before offering each meal daily, and 15% of fresh fecal samples were acidified with 10 mL of 10% (*v*/*v*) H_2_SO_4_ to prevent nitrogen loss. The daily urine output was collected in a plastic container containing 20 mL of H_2_SO_4_ (500 mL/L) to ensure ammonia preservation, and samples were pooled and then stored at −20 °C for future determination.

On d 0 and d 30 of the collection period, blood samples (10 mL) were collected from the jugular veins of all sheep into heparinized tubes. Subsequently, the blood samples were centrifuged at 3000× *g* for 15 min at 4 °C to obtain plasma supernatants, and then the collected plasma samples were stored at −20 °C until analysis.

### 5.5. Laboratory Analysis

Samples of individual feed ingredients, orts, and feces were dried in an oven at 55 °C for 48 h, ground to pass a 1 mm screen (Wiley Mill; Arthur A. Thomas Co., Denver, CO, USA), and analyzed for dry matter (DM; 934.01), crude protein (CP; 976.05), ether extract (EE; 920.39), and ash (942.05) according to AOAC [52]. The neutral detergent fiber (NDF) content was analyzed with heat-stable α-amylase and sodium sulfite using the procedure described by Van Soest et al. [53]. The acid detergent fiber (ADF) content was analyzed according to AOAC [54].

Plasma samples were sent to Heilongjiang Electric Power Hospital (Harbin, China) for analysis of total protein (TP), albumin (ALB), alanine amino transferase (ALT), aspartate transaminase (AST), alkaline phosphatase (ALP), total bilirubin (TBIL), triglyceride (TG), cholesterol (TC), urea nitrogen (BUN), and glucose (Glu). Samples were analyzed with an automatic biochemical analyzer (Roche Cobus Mira Plus, Cham, Switzerland) using commercial diagnostic kits (Nanjing Jian Cheng Bioengineering Institute; Nanjing, China). The concentrations of glutathione peroxidase (GSH-Px), superoxide dismutase (SOD), total antioxidant capacity (T-AOC), malondialdehyde (MDA), immunoglobin A (IgA), immunoglobin M (IgM), immunoglobin G (IgG), interleukin-2 (IL-2), interleukin-4 (IL-4), and interleukin-6 (IL-6) were measured using ELISA kits (Nanjing Jian Cheng Bioengineering Institute; Nanjing, China).

### 5.6. Statistical Analysis

The results from Experiment 1 were analyzed using SPSS 20.0 software for ANOVA and Duncan’s multiple comparison. Excel 2016 was used for data processing. The results from Experiment 2 were analyzed using the MIXED procedure of SAS (version 9.2; SAS Institute Inc., Cary, NC, USA). Data on d 0 were used as a covariate for analysis on d 30. The statistical model was as follows: *Y_ij_* = *µ* + *α_i_* + *β* (*X_ij_* − ${\bar{X}}_{i}$) + *E_ij_*, where *Y_ij_* is the dependent variable, *µ* is the overall mean, *α_i_* is the treatment effect, *E_ij_* is the residual error, *X_ij_* is the covariant of *Y_ij_*, $\bar{X}$ is the mean value of *X_ij_*, and *β* is the regression coefficient of *Y_ij_* on *X_ij_*. Significant differences were declared at *p* ≤ 0.05, and trends were defined at 0.05 < *p* ≤ 0.10.

## Figures and Tables

**Table 1:** Effects of different ammoniation treatment conditions on AFB1 content in the diet.

Group Numbers	MPa	h	AFB1 (μg/kg)
I	0	0	2550
II	0.1	1	647.50 ± 3.54
III	0.1	2	562.50 ± 3.54
IV	0.1	3	467.00 ± 4.24
V	0.2	1	630.50 ± 0.71
VI	0.2	2	502.50 ± 0.71
VII	0.2	3	211.55 ± 0.64
VIII	0.3	1	529.50 ± 6.35
IX	0.3	2	485.00 ± 7.07
X	0.3	3	65.90 ± 7.50
			Main effect
Pressure (mPa)	0		2550.00 ^a^
	0.1		559.00 ^b^
	0.2		448.18 ^c^
	0.3		360.13 ^c^
Time (h)	0		2500.00 ^a^
	1		602.50 ^b^
	2		516.67 ^b^
	3		248.15 ^c^
			*p*-value
Pressure			<0.01
Time			<0.01
Pressure × Time			0.04

Note: ^a,b,c^ Means within a row with different superscripts differ (*p* < 0.05).

**Table 2:** Effects of ammoniated AFB1 diet on growth performance and nutrient digestibility in sheep.

	Treatment ^2^				
Item ^1^	C	T	AT	SEM	*p*-Value
ADG (g/d)	86.43	84.12	85.69	3.98	0.08
DMI (g/d)	1270 ^a^	1188 ^b^	1265 ^a^	12.92	<0.01
FCR	14.82	14.21	14.79	1.37	0.43
Nutrient digestibility, %					
DM	63.59 ^a^	58.97 ^b^	63.38 ^a^	0.70	0.02
CP	72.35 ^b^	66.91 ^c^	76.33 ^a^	0.71	<0.01
EE	73.95 ^a^	67.98 ^b^	74.25 ^a^	1.17	0.02
NDF	47.43	47.38	46.89	0.98	0.13
ADF	31.33	33.69	33.80	0.85	0.10

^a,b,c^ Means within a row with different superscripts differ (*p* < 0.05). ^1^ ADG—average daily gain; DMI—dry matter intake; FCR—feed conversion ratio; DM—dry matter; EE—ether extract; CP—crude protein; NDF—neutral detergent fiber; ADF—acid detergent fiber. ^2^ C—control; T—aflatoxin diet containing C and 75 μg of AFB1/kg; AT—ammoniated diet containing C and 75 μg of AFB1/kg.

**Table 3:** Effects of the ammoniated AFB1 diet on the nitrogen utilization of sheep.

	Treatment ^2^				
Item ^1^	C	T	AT	SEM	*p*-Value
N intake, g/d	23.62 ^b^	22.04 ^c^	24.89 ^a^	0.21	0.03
Fecal N excretion g/d	5.97 ^b^	5.91 ^b^	6.58 ^a^	0.18	0.02
Fecal N excretion (g/kg of N intake)	252 ^b^	268 ^a^	263 ^a^	9.13	0.04
Urinary N excretion g/d	8.30 ^b^	9.78 ^a^	8.29 ^b^	0.17	<0.01
Urinary N excretion (g/kg of N intake)	366 ^b^	443 ^a^	335 ^b^	7.12	<0.01
Retained N g/d	9.35 ^a^	6.35 ^b^	9.54 ^a^	0.43	<0.01
Retained N (g/kg of N intake)	397 ^a^	290 ^b^	387 ^a^	10.12	0.03

^a,b,c^ Means within a row with different superscripts differ (*p* < 0.05). ^1^ N—nitrogen. ^2^ C—control; T—aflatoxin diet containing C and 75 μg of AFB1/kg; AT—ammoniated diet containing C and 75 μg of AFB1/kg.

**Table 4:** Effects of the ammoniated AFB1 diet on sheep biochemical indices.

	Treatment ^2^				
Item ^1^	C	T	AT	SEM	*p*-Value
TP (g/L)	66.06 ^a^	63.66 ^b^	66.10 ^a^	1.19	<0.01
ALB (g/L)	33.48 ^a^	28.82 ^b^	33.26 ^a^	0.60	<0.01
ALT (U/L)	24.60 ^b^	30.60 ^a^	24.40 ^b^	1.04	<0.01
AST (U/L)	120.00 ^b^	156.20 ^a^	123.40 ^b^	3.11	0.02
ALP (U/L)	155.20	160.80	158.00	3.99	0.52
TG (mmol/L)	0.51	0.52	0.55	0.07	0.18
TC (mmol/L)	2.48	2.69	2.36	0.21	0.83
TBIL (μmol/L)	1.98 ^b^	2.68 ^a^	1.90 ^b^	0.13	<0.01
BUN (mmol/L)	7.32	6.26	6.98	0.40	0.17
Glu (mmol/L)	3.92	3.54	3.64	0.21	0.38

^a,b^ Means within a row with different superscripts differ (*p* < 0.05). ^1^ TP—total protein; ALB—albumin; ALT—alanine amino transferase; AST—aspartate transaminase; ALP—alkaline phosphatase; TBIL—total bilirubin; TG—triglyceride; TC—cholesterol; BUN—urea nitrogen; Glu—glucose. ^2^ C—control; T—aflatoxin diet containing C and 75 μg of AFB1/kg; AT—ammoniated diet containing C and 75 μg of AFB1/kg.

**Table 5:** Effects of ammoniated AFB1 diet on antioxidant and immune indices of sheep.

	Treatment ^2^				
Item ^1^	C	T	AT	SEM	*p*-Value
SOD (U/mL)	67.62 ^a^	55.09 ^b^	63.61 ^a^	1.94	<0.01
MDA (mmol/mL)	2.18 ^b^	3.08 ^a^	2.14 ^b^	0.36	<0.01
GSH-PX (U/mL)	92.75 ^a^	60.59 ^b^	90.13 ^a^	2.76	0.04
T-AOC (U/mL)	0.61 ^a^	0.51 ^b^	0.60 ^a^	0.03	0.03
IgA (mg/mL)	3.57 ^a^	2.50 ^b^	3.00 ^a^	0.23	<0.01
IgG (mg/mL)	12.10 ^a^	9.91 ^b^	12.92 ^a^	0.79	<0.01
IgM (mg/mL)	1.66 ^a^	1.29 ^b^	1.54 ^a^	0.11	<0.01
IL-2 (ng/L)	40.79	38.45	39.84	2.91	0.24
IL-4 (ng/L)	89.94 ^a^	80.95 ^b^	90.12 ^a^	3.43	<0.01
IL-6 (ng/L)	116.86 ^b^	135.60 ^a^	112.36 ^b^	2.09	<0.01

^a,b^ Means within a row with different superscripts differ (*p* < 0.05). ^1^ SOD—superoxide dismutase; MDA—malondialdehyde; GSH-PX—glutathione peroxidase; T-AOC—total antioxidation capacity; IgA—immunoglobulin A; IgG—immunoglobulin G; IgM—immunoglobulin M; IL-2—interleukin-2; IL-4—interleukin-4; IL-6—interleukin-6. ^2^ C—control; T—aflatoxin diet containing C and 75 μg of AFB1/kg; AT—ammoniated diet containing C and 75 μg of AFB1/kg.

**Table 6:** Dietary compositions and nutrition levels (DM basis).

	Treatments ^2^		
Items ^1^	C	T	AT
Ingredient, % of DM			
Chinese wild grass hay	60.0	60.0	60.0
Corn	23.1	23.1	23.1
Soybean meal	13.4	13.4	13.4
NaCl	1.5	1.5	1.5
CaHPO_4_	0.5	0.5	0.5
Limestone	0.5	0.5	0.5
Premix ^3^	1.0	1.0	1.0
Chemical composition, % of DM			
DM	90.3	90.3	90.9
OM	94.4	94.3	94.2
CP	11.5	11.6	12.3
EE	3.2	3.3	3.3
NDF	42.3	42.6	42.5
ADF	26.8	26.2	26.1

^1^ DM—dry matter; OM—organic matter; CP—crude protein; EE—ether extract; NDF—neutral detergent fiber; ADF—acid detergent fiber. ^2^ C—control; T—aflatoxin diet containing C and 75 μg of AFB1/kg; AT—ammoniated diet containing C and 75 μg of AFB1/kg. ^3^ Mineral elements per kilogram of dry matter are Cu 12 mg, Mn 42 mg, Zn 34 mg, Se 0.2 mg, I 0.6 mg, Co 0.2 mg, vitamin A 3500 IU, vitamin D 1300 IU, and vitamin E 30 IU.

## Data Availability

The data presented in this study are available in this article.

## References

[1] Mahmood S., Younus M., Aslam A., Anjum A., Sohail M. (2017). Chemical detoxification of AFB1 in experimental quails using commercially available toxin binders. J. Anim. Plant. Sci..

[2] Wang Y., Liu F., Liu M., Zhou X., Wang M., Cao K., Jin S., Shan A., Feng X. (2022). Curcumin mitigates aflatoxin B1-induced liver injury via regulating the NLRP3 inflammasome and Nrf2 signaling pathway. Food Chem. Toxicol..

[3] Queiroz O.C.M., Han J.H., Staples C.R., Adesogan A.T. (2012). Effect of adding a mycotoxin-sequestering agent on milk aflatoxin M1 concentration and the performance and immune response of dairy cattle fed an aflatoxin B1-contaminated diet. J. Dairy Sci..

[4] Yu L., Zhang Y., Hu C., Wu H., Yang Y., Huang C., Jia N. (2015). Highly sensitive electrochemical impedance spectroscopy immunosensor for the detection of AFB1 in olive oil. Food Chem..

[5] Zhang L.Y., Liu S., Zhao X.J., Wang N., Jiang X., Xin H.S., Zhang Y.G. (2019). Lactobacillus rhamnosus GG modulates gastrointestinal absorption, excretion patterns, and toxicity in Holstein calves fed a single dose of aflatoxin B1. J. Dairy Sci..

[6] Eaton D.L., Groopman J.D. (1994). The Toxicology of Aflatoxins: Human Health, Veterinary, and Agricultural Significance.

[7] Yener Z., Celik I., Ilhan F., Bal R. (2009). Effects of Urtica dioica L. seed on lipid peroxidation, antioxidants and liver pathology in aflatoxin-induced tissue injury in rats. Food Chem. Toxicol..

[8] Astoreca A., Vaamonde G., Dalcero A., Ramos A., Marín S. (2012). Modelling the effect of temperature and water activity of Aspergillus flavus isolates from corn. Int. J. Food Microbiol..

[9] Bryden W.L. (2012). Mycotoxin contamination of the feed supply chain: Implications for animal productivity and feed security. Anim. Feed Sci. Technol..

[10] Damiano S., Jarriyawattanachaikul W., Girolami F., Longobardi C., Nebbia C., Andretta E., Lauritano C., Dabbou S., Avantaggiato G., Schiavone A. (2022). Curcumin Supplementation Protects Broiler Chickens Against the Renal Oxidative Stress Induced by the Dietary Exposure to Low Levels of Aflatoxin B1. Front. Vet. Sci..

[11] Fu J.C., Chen Q., Du J., Shi B.M., Shan A.S. (2013). Effectiveness of maifanite in reducing the detrimental effects of aflatoxin B1 on hematology, aflatoxin B1 residues, and antioxidant enzymes activities of weanling piglets. Livest. Sci..

[12] Ogunade I., Arriola K., Jiang Y., Driver J., Staples C., Adesogan A. (2016). Effects of 3 sequestering agents on milk aflatoxin M1 concentration and the performance and immune status of dairy cows fed diets artificially contaminated with aflatoxin B1. J. Dairy Sci..

[13] Fernández A., Ramos J., Saez T., Sanz M., Verde M. (1995). Changes in the coagulation profile of lambs intoxicated with aflatoxin in their feed. Veter. Res..

[14] Fernández A., Ramos J.J., Sanz M.D.C., Saez T., De Luco D.F. (1996). Alterations in the Performance, Haematology and Clinical Biochemistry of Growing Lambs Fed with Aflatoxin in the Diet. J. Appl. Toxicol..

[15] Ramos J., Fernández A., Sáez T., Sanz M., Marca M. (1996). Effect of aflatoxicosis on blood mineral constituents of growing lambs. Small Rumin. Res..

[16] Jouany J.P. (2007). Methods for preventing, decontaminating and minimizing the toxicity of mycotoxins in feeds. Anim. Feed Sci. Technol..

[17] ParK D.L., Lee L.S., Price R.L., Pohland A.E. (1988). Review of the Decontamination of Aflatoxins by Ammoniation: Current Status and Regulation. J. AOAC Int..

[18] Weng C.Y., Martinez A.J., Park D.L. (1994). Efficacy and permanency of ammonia treatment in reducing aflatoxin levels in corn. Food Addit. Contam..

[19] Hao X.Z., Yue X.Y., Chang-Bin L.I., Xin T., Jun-Xia L.I. (2015). Study on the degradation of aflatoxin B1 by Stenotrophomonas sp.42-2. Sci.Tech. Food Ind..

[20] Allameh A., Safamehr A., Mirhadi S.A., Shivazad M., Razzaghi-Abyaneh M., Afshar-Naderi A. (2005). Evaluation of biochemical and production parameters of broiler chicks fed ammonia treated aflatoxin contaminated maize grains. Anim. Feed Sci. Technol..

[21] Nyandieka H.S., Maina J.O., Nyamwange C. (2014). Destruction of aflatoxins in contaminated maize samples using ammoniation procedures. East Cent. Afr. J. Pharm. Sci..

[22] Norred W.P. (1982). Ammonia Treatment to Destroy Aflatoxins in Corn. J. Food Prot..

[23] Boonyaratpalin M., Supamattaya K., Verakunpiriya V., Suprasert D. (2015). Effects of aflatoxin B1 on growth performance, blood components, immune function and histopathological changes in black tiger shrimp (*Penaeus monodon Fabricius*). Aquacult. Res..

[24] Deng S.X., Tian L.X., Liu F.J., Jin S.J., Liang G.Y., Yang H.J., Du Z.Y., Liu Y.J. (2010). Toxic effects and residue of aflatoxin B1 in tilapia (*Oreochromis niloticus × O. aureus*) during long-term dietary exposure. Aquaculture..

[25] Dersjant-Li Y., Verstegen M.W.A., Gerrits W.J.J. (2003). The impact of low concentrations of aflatoxin, deoxynivalenol or fumonisin in diets on growing pigs and poultry. Nutr. Res. Rev..

[26] He J., Zhang K., Chen D., Ding X., Feng G., Ao X. (2013). Effects of maize naturally contaminated with aflatoxin B1 on growth performance, blood profiles and hepatic histopathology in ducks. Livest. Sci..

[27] Kocabas C.N., Coşkun T., Yurdakök M., HazwrogÆlu R. (2003). The effects of aflatoxin B1 on the development of kwashiorkor in mice. Hum. Exp. Toxicol..

[28] Yarru L., Settivari R., Gowda N., Antoniou E., Ledoux D., Rottinghaus G. (2009). Effects of turmeric (Curcuma longa) on the expression of hepatic genes associated with biotransformation, antioxidant, and immune systems in broiler chicks fed aflatoxin. Poult. Sci..

[29] Pu J., Yuan Q., Yan H., Tian G., Chen D., He J., Zheng P., Yu J., Mao X., Huang Z. (2021). Effects of chronic exposure to low levels of dietary aflatoxin b1 on growth performance, apparent total tract digestibility and intestinal health in pigs. Animals.

[30] Kermanshahi H.L., Akbari M.R., Maleki M. (2007). Effect of prolonged low level Inclusion of Aflatoxin B, into diet on performance, Nutrent Digestibil. J. Anim. Vet. Adv..

[31] Yang Z.B., Wan X.L., Yang W.R., Jiang S.Z., Zhang G.G., Johnston S.L., Chi F. (2014). Effects of naturally mycotoxin-contaminated corn on nutrient and energy utilization of ducks fed diets with or without Calibrin-A. Poult. Sci..

[32] Wan X.L., Yang Z.B., Yang W.R., Jiang S.Z., Zhang G.G., Johnston S.L., Chi F. (2013). Toxicity of increasing aflatoxin B1 concentrations from contaminated corn with or without clay adsorbent supplementation in ducklings. Poult. Sci..

[33] Dang H., Obitsu T., Sugino T. (2018). Effects of ensiling treatment for tuber crop forages and grain source on carbohydrate digestion, nitrogen utilization, and urea metabolism in sheep. Anim. Feed Sci. Technol..

[34] Huhtanen P., Hristov A.N. (2009). A meta-analysis of the effects of dietary protein concentration and degradability on milk protein yield and milk N efficiency in dairy cows. J. Dairy Sci..

[35] Lynch G., Smith D., Covey F., Gordon C. (1973). Aflatoxin and Nitrogen Balance in the Young Calf. J. Dairy Sci..

[36] Xiong J., Wang Y., Nennich T., Li Y., Liu J. (2015). Transfer of dietary aflatoxin B1 to milk aflatoxin M1 and effect of inclusion of adsorbent in the diet of dairy cows. J. Dairy Sci..

[37] Solis-Cruz B., Hernandez-Patlan D., Petrone V.M., Pontin K.P., Latorre J.D., Beyssac E., Hernandez-Velasco X., Merino-Guzman R., Arreguin M.A., Hargis B.M. (2019). Evaluation of a Bacillus-based direct-fed microbial on aflatoxin B1 toxic effects, performance, immunologic status, and serum biochemical parameters in broiler chickens. Avian Dis..

[38] Klein P.J., Van Vleet T.R., Hall J.O., Coulombe R.A. (2002). Biochemical factors underlying the age-related sensitivity of turkeys to aflatoxin B1. Comp. Biochem. Physiol. Part C Toxicol. Pharmacol..

[39] Harvey R.B., Kubena L., Phillips T., Huff W., Corrier D. (1989). Prevention of aflatoxicosis by addition of hydrated sodium calcium aluminosilicate to the diets of growing barrows. Am. J. Veter. Res..

[40] Gao S., Zhang L., Zhu D., Huang J., Yang J., Jiang J., Wu H., Lv G. (2021). Effects of glucose oxidase and bacillus subtilis on growth performance and serum biochemical indicexs of broilers exposed to aflatoxin B1 and endotoxin. Anim. Feed Sci. Technol..

[41] Sordillo L.M. (2013). Selenium-Dependent Regulation of Oxidative Stress and Immunity in Periparturient Dairy Cattle. Veter. Med. Int..

[42] Shi Y.H., Xu Z.R., Feng J.L., Wang C.Z. (2006). Efficacy of modified montmorillonite nanocomposite to reduce the toxicity of aflatoxin in broiler chicks. Anim. Feed Sci. Technol..

[43] Abdel-Wahhab M.A., Hasan A.M., Aly S.E., Mahrous K.F. (2005). Adsorption of sterigmatocystin by montmorillonite and inhibition of its genotoxicity in the Nile tilapia fish (Oreachromis nilaticus). Mutat. Res. Toxicol. Environ. Mutagen..

[44] Long M., Zhang Y., Li P., Yang S.-H., Zhang W.-K., Han J.-X., Wang Y., He J.-B. (2016). Intervention of Grape Seed Proanthocyanidin Extract on the Subchronic Immune Injury in Mice Induced by Aflatoxin B1. Int. J. Mol. Sci..

[45] Mohajeri M., Behnam B., Cicero A.F.G., Sahebkar A. (2017). Protective effects of curcumin against aflatoxicosis: A comprehensive review. J. Cell. Physiol..

[46] Hill N., Sarvetnick N. (2002). Cytokines: Promoters and dampeners of autoimmunity. Curr. Opin. Immunol..

[47] Dou X., Yan D., Ma Z., Gao N., Shan A. (2022). Sodium butyrate alleviates LPS-induced kidney injury via inhibiting TLR2/4 to regulate rBD2 expression. J. Food Biochem..

[48] Dey D.K., Chang S.N., Kang S.C. (2021). The inflammation response and risk associated with aflatoxin B1 contamination was minimized by insect peptide CopA3 treatment and act towards the beneficial health outcomes. Environ. Pollut..

[49] Muhammad I., Wang X., Li S., Li R., Zhang X. (2018). Curcumin confers hepatoprotection against AFB 1-induced toxicity via activating autophagy and ameliorating inflammation involving Nrf2/HO-1 signaling pathway. Mol. Biol. Rep..

[50] Ellinger-Ziegelbauer H., Stuart B., Wahle B., Bomann W., Ahr H.J. (2005). Comparison of the expression profiles induced by genotoxic and nongenotoxic carcinogens in rat liver. Mutat. Res. Mol. Mech. Mutagen..

[51] Qian G., Tang L., Guo X., Wang F., Massey M.E., Su J., Guo T.L., Williams J.H., Phillips T.D., Wang J.-S. (2013). Aflatoxin B_1_modulates the expression of phenotypic markers and cytokines by splenic lymphocytes of male F344 rats. J. Appl. Toxicol..

[52] AOAC (1990). Official Methods of Analysis: Changes in Official Methods of Analysis Made at the Annual Meeting.

[53] Van Soest P.V., Robertson J., Lewis B. (1991). Methods for dietary fiber, neutral detergent fiber, and nonstarch polysaccharides in relation to animal nutrition. J. Dairy Sci..

[54] AOAC (2000). Official Methods of Analysis.

